# Negative allosteric regulation of protein arginine methyltransferase 1 (PRMT1) through half-of-sites reactivity

**DOI:** 10.1016/j.jbc.2026.113425

**Published:** 2026-08-12

**Authors:** Vincent Z. Rossi, Robert S. Wilson-Kovacs, Yuan Gao, Caroline Velez, Sofiia Hakh, Chao Xue, Jun Qu, Vicki Wysocki, Orlando Acevedo, Joan M. Hevel

**Affiliations:** 1 Department of Chemistry and Biochemistry, Utah State University, Logan, Utah, USA; 2 Department of Chemistry, University of Miami, Coral Gables, Florida, USA; 3 School of Chemistry & Biochemistry, Georgia Institute of Technology, Atlanta, Georgia, USA; 4 Department of Pharmaceutical Sciences, University at Buffalo, Buffalo, New York, USA

**Keywords:** protein arginine methyltransferase (PRMT), negative allostery, arginine methylation, subunit communication, half-of-sites, allosteric regulation, enzyme catalysis, enzyme mechanism, oligomerization, post-translational modification (PTM)

## Abstract

Protein arginine methyltransferase 1 (PRMT1) activity is essential for maintaining proper eukaryotic cellular function; however, its dysregulation contributes to the progression of several human diseases and cancers, making PRMT1 an attractive therapeutic target. Efforts to inhibit PRMT1 through active-site-directed competitive strategies have faced significant challenges, highlighting the need to discover and exploit allosteric regulatory mechanisms. We hypothesized that PRMT1 dimerization enables communication between individual PRMT1 subunits, which modulates catalytic activity. Consistent with this idea, molecular dynamics simulations found that chemical reactivity was productive only when a single active site within the PRMT1 dimer was occupied by peptide substrate, suggesting that PRMT1 exhibits half-of-sites reactivity. To validate this experimentally, we engineered a model PRMT1 dimer with only a single functional active site using dimeric *Caenorhabditis elegans* PRMT1 (cePRMT1). *In vitro* kinetic characterization showed that the single active site mutant enzyme exhibits ∼90% of the maximal activity of the wild-type enzyme, supporting that PRMT1 is half-of-sites reactive. Additionally, native mass spectrometry showed that the half-of-sites reactivity in the cePRMT1 dimer is driven by negative cooperative binding for peptide substrate. Together, these findings expand our mechanistic understanding of inter-subunit communication in PRMT1 oligomers and identify half-of-sites reactivity as a previously unrecognized regulatory feature that may be leveraged for the development of PRMT1-selective allosteric inhibitors.

Protein arginine methylation is a highly prevalent post-translational modification rivaling modifications such as ubiquitination and phosphorylation in predominance (1, 2). In eukaryotic cells, the methylation of an estimated 0.5% to 4% of arginine residues in both histone and non-histone proteins (1, 3) plays a fundamental role in regulating a wide range of cellular processes across all tissue types (4, 5, 6). Arginine methylation is catalyzed by a family of enzymes called protein arginine methyltransferases (PRMTs), which perform the transfer of one or two methyl groups to the guanidino nitrogen of arginine residues in protein substrates using S-adenosyl-L-methionine (SAM) as the methyl donor, producing the methylated protein and S-adenosyl-homocysteine (SAH) as a result. The mammalian PRMT family consists of nine members, of which PRMT1 is the predominant isoform and is reported to perform an estimated 85% of total protein arginine methylation (7). PRMT1 is a type I PRMT meaning it can form both monomethyl arginine (MMA) and asymmetric dimethylarginine (ADMA) at substrate arginine residues in proteins.

PRMT1 methyltransferase activity is crucial for maintaining proper cellular health and function, with substantial evidence linking PRMT1 dysregulation to the progression of cardiovascular (8), neurodegenerative (9), immune-related (10, 11), and chronic liver (12) diseases. Additionally, abnormal increases in PRMT1 mRNA and protein expression levels have been associated with several forms of cancer (13, 14, 15, 16, 17), and recent studies have identified relationships between specific PRMT1 methylation events and cancer metastasis (13, 15). The association of aberrant PRMT1-mediated arginine methylation and various disease states positions PRMT1 as an appealing target in therapeutic intervention and has prompted intense efforts devoted to developing PRMT1-specific inhibitors (18, 19). Unfortunately, only two type I PRMT inhibitors have advanced to clinical trials (https://clinicaltrials.gov/study/NCT06224387, https://clinicaltrials.gov/study/NCT03666988) (20), neither of which have successfully progressed through clinical development or specifically target PRMT1 over other type I PRMTs. Many type I PRMT inhibitors to date are designed to target structural features of the PRMT active site, such as the SAM-binding pocket, putative substrate binding surfaces, or a combination of both (18). While the field has recently made significant strides in designing these inhibitor classes, the highly conserved nature of the PRMT active site suggests that competitive inhibitors may not be the most effective avenue for PRMT1-specific inhibition. This calls for an alternative direction for inhibitor design to target specific PRMT isoforms, such as those that manipulate unique allosteric regulatory mechanisms within the enzyme (21, 22, 23, 24). However, our current understanding of the mechanistic role distal elements play in PRMT1 catalysis remains limited to only a few studies (25, 26) and requires further elucidation.

One characteristic of PRMT1 activation that has potential to be exploited and has recently garnered our attention is PRMT1 homo-oligomerization. For many enzymes, oligomerization provides new opportunities for regulatory control accomplished through communication between individual subunits (27, 28, 29). Even though each PRMT1 subunit harbors what appears to be a complete active site, the minimal catalytically competent oligomeric unit of PRMT1 is the homodimer (30, 31, 32), indicating that oligomerization is a crucial characteristic of the enzyme. Additionally, our group (32) along with others (33, 34), has recently demonstrated that PRMT1 oligomerization serves as a mechanism to regulate enzymatic activity. However, whether individual subunits within PRMT1 oligomers engage in allosteric inter-subunit communication to further regulate catalysis remains poorly understood.

In this study, molecular dynamics simulations found that chemical reactivity in the human PRMT1 dimer was only productive when a single active site within the dimer was occupied by peptide substrate, suggesting that PRMT1 exhibits half-of-sites reactivity. To address this experimentally, we designed a model system to investigate how the presence of two active sites influences PRMT1 product formation. Using a naturally dimeric ortholog of PRMT1 from *Caenorhabditis elegans*, denoted cePRMT1, we designed and isolated a unique PRMT1 dimer that contains only one catalytically functional active site. Using this model system, we show that a PRMT1 dimer with only one catalytically competent active site has nearly equivalent activity of a PRMT1 dimer with two competent active sites. Additionally, we show that a cePRMT1 dimer binds its substrates in a 1:1:2 dimer:peptide substrate:cofactor ratio along the reaction coordinate, consistent with a negative cooperativity mechanism for substrate binding. Lastly, we show that a cePRMT1 dimer with a single functional active site can asymmetrically dimethylate arginine residues. Overall, our work demonstrates that inter-subunit communication within the PRMT1 dimer allosterically regulates enzymatic activity, deepening our understanding of the regulatory complexity conferred through PRMT1 oligomerization and unveiling an exploitable fine-tuning mechanism that can be leveraged to guide sophisticated PRMT1 inhibitor design.

## Results

### Molecular dynamics simulations identify asymmetry in the PRMT1 dimer

**Figure 1:**
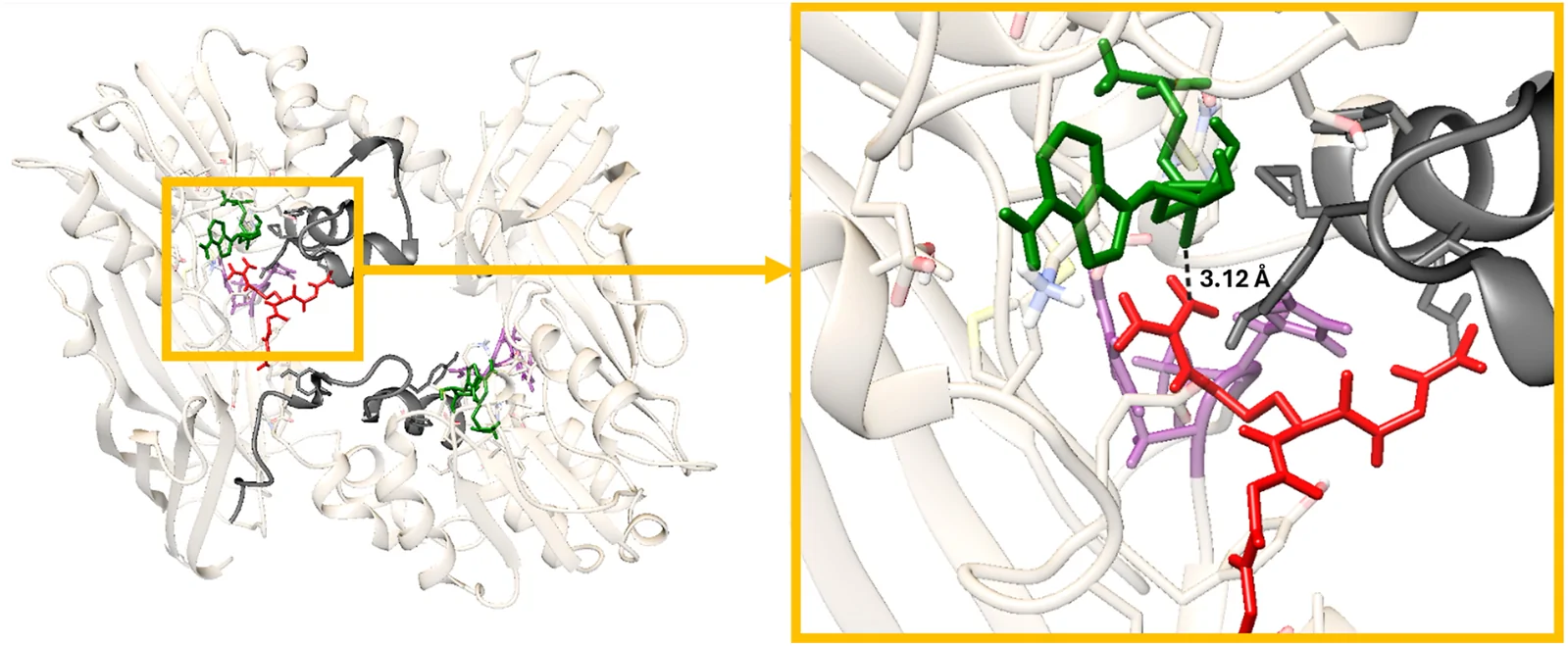
**An illustrated structure of a PRMT1 dimer bound asymmetrically with a single GGRG peptide (*red*) and two SAM cofactors (*green*).** The THW loop is shown in *purple* and the *N*-termini in *black*.

Our group has previously demonstrated that molecular dynamics (MD) simulations of monomeric PRMT1 can successfully predict catalytic activity and product specificity during the first and second methylation turnovers (35). These studies identified key hydrogen-bonding networks, active-site geometries, and donor–acceptor distances that serve as structural metrics for distinguishing active wild-type and mutant enzymes. More recently, however, both our work (31, 32) and that of others (30, 36) established that PRMT1 oligomerization is required for catalytic activity, prompting us to investigate whether incorporation of the catalytically relevant dimeric architecture would alter the mechanistic features observed in MD simulations of the monomer. Initial simulations employing a symmetric PRMT1 dimer containing two bound GGRG peptides and two SAM cofactors proved unstable, with rapid expulsion of one peptide from its active site, and the other peptide exhibiting poor binding geometry. In contrast, simulations of an asymmetric dimer containing a single bound GGRG peptide and two SAM molecules remained stable and evolved toward an activated catalytic configuration (Fig. 1). Examination of the probability of forming a near-attack conformer resembling the transition state revealed a highly organized active site in which the peptide substrate and SAM adopt an optimal catalytic arrangement for S_N_2 methyl transfer (see Table S1 and Figs. S1–S3). Collectively, these MD results suggest functional asymmetry and potential half-of-sites reactivity (37, 38) within the PRMT1 dimer, motivating the experimental studies described below to directly evaluate this mechanistic hypothesis.

### A *C. elegans* PRMT1 model allows for isolation of a PRMT1 dimer with a single functional active site

We sought to validate PRMT1 half-of-sites reactivity by engineering a PRMT1 dimer containing only a single functional active site, an established strategy for interrogating homo-oligomeric enzymes (39, 40, 41). Under this negative allostery model, the single-active-site dimer should show activity nearly equivalent to the two-active-site dimer. However, implementing this approach with human PRMT1 was challenged by its propensity to form tetramers at physiologically relevant enzyme concentrations (32). To ensure a dimeric enzyme system, we selected the *C. elegans* orthologue of PRMT1 (cePRMT1) as the basis for our design. Our group has shown that unlike human PRMT1, cePRMT1 is predominantly dimeric in a nanomolar to low micromolar range (Fig. S5). Because cePRMT1 shares ∼68% sequence identity and ∼78% sequence similarity with the human enzyme, it is an ideal candidate for studying PRMT1 activity in a dimeric context.

**Figure 2:**
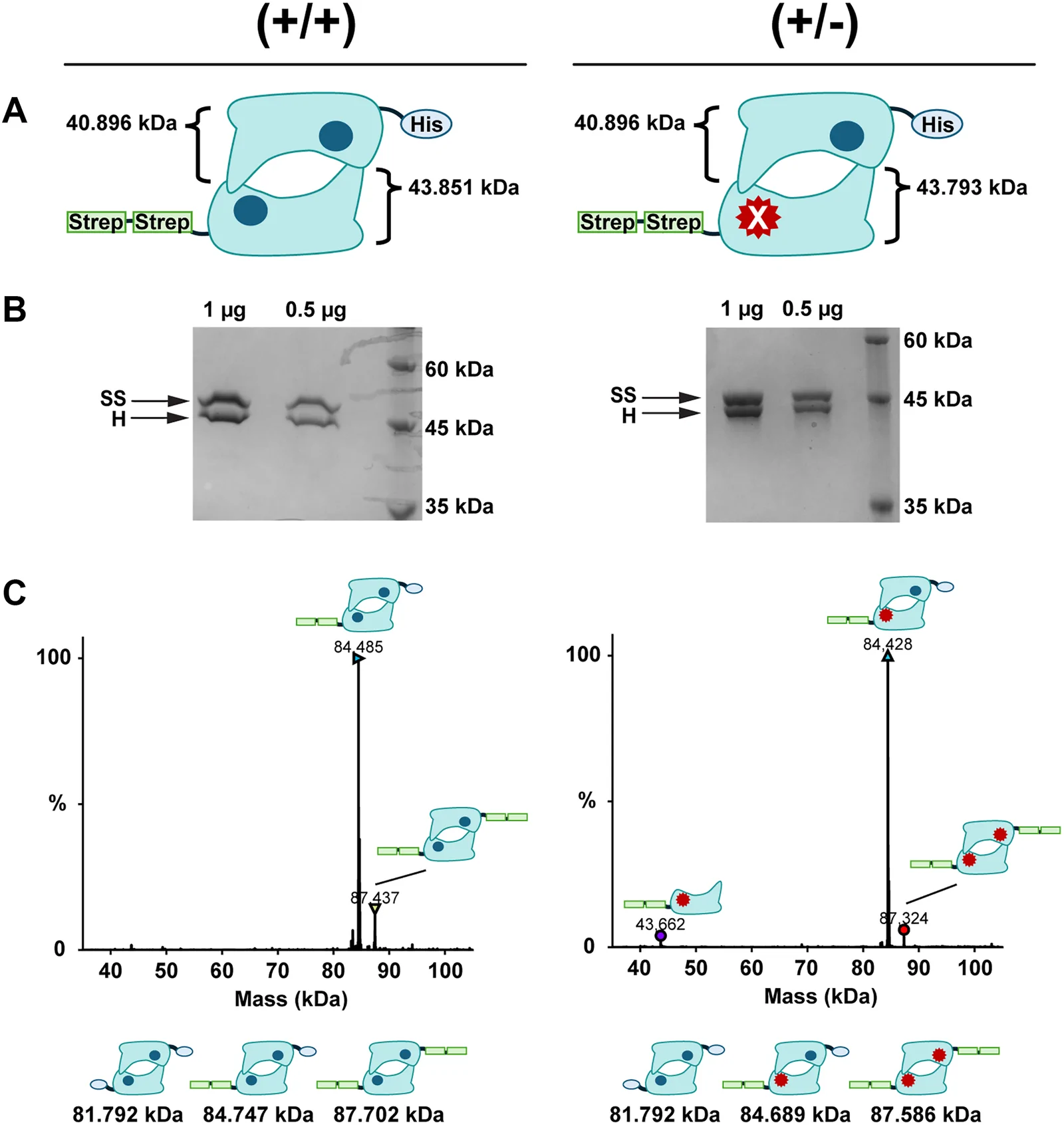
**CePRMT1 heterodimer design and evaluation.***A*, cartoon depicting the design of the (+/+) and (+/−) cePRMT1 heterodimers. Functional active sites are shown in *blue*, while the “-“ catalytically dead active site is shown in *red* and denoted with an “X”. Subunits with the two *lime green rectangles* represent 2xstrep-tagged subunits, while subunits with the single *light blue* oval represent his-tagged subunits. *B*, various amounts of the (+/+) and (+/−) heterodimers were loaded onto an SDS PAGE gel and showed a 1:1 band intensity for the his-tagged (*bottom band*, H) and 2xstrep-tagged (*top band*, SS) subunits. Gels were stained with Coomassie *Blue* and band intensity was quantified with ImageLab 5.1 software using the lanes and bands tool. The percentage of 2xstrep-tagged subunit quantified though gel densitometry was 53 ± 1% for the (+/−) and 52 ± 2% for the (+/+) heterodimer. Uncropped images of each gel are provided in Figure S7. *C*, native MS performed at 1 μM enzyme (based on the expected heterodimer) shows that the predominant species for the (+/−) and (+/+) is the heterodimer. Experimentally determined masses are consistent with each subunit experiencing loss of the initiator methionine. Enzyme samples were buffer exchanged into 150 mM ammonium acetate, adjusted to pH 7.6 to match the kinetic assay conditions, diluted to 1 μM (dimer concentration), and immediately injected into the mass spectrometer.

Considering this, we developed two cePRMT1 heterodimers. In this context, “heterodimer” refers to a dimer with a differential purification tag built into each cePRMT1 subunit. The first heterodimer consisted of one fully functional his-tagged subunit and one fully functional 2xstrep-tagged subunit (+/+ cePRMT1) (Fig. 2*A*). The second heterodimer consisted of one fully functional his-tagged subunit and the loss-of-function mutation glutamate 153 (human orthologue residue numbering) to alanine (E153A mutant) in the 2xstrep-tagged subunit. This would result in a catalytically inactive subunit dimerizing with a fully functional one ( +/− cePRMT1) (Fig. 2*A*). This mutation was selected for the inactive subunit design since it has been shown to abolish enzymatic activity in human PRMT1 while maintaining oligomeric integrity (32, 42) (Fig. S6). Isolation of the his/2xstrep heterodimeric enzymes was achieved through use of a streptavidin affinity column in tandem with an immobilized metal affinity column. SDS PAGE gel densitometry showed that the purification yielded a 1:1 stoichiometry of the 2xstrep-tagged and his-tagged subunits (Figs. 2*B* and S7). Additionally, native mass spectrometry (native MS) performed at 1 μM protein (dimer concentration) confirmed that the purification strategy results in a dimeric population that is almost entirely the heterodimeric cePRMT1 species (Fig. 2*C*).

### Kinetic analysis of the *C. elegans* PRMT1 heterodimers is consistent with half-of-sites reactivity

**Figure 3:**
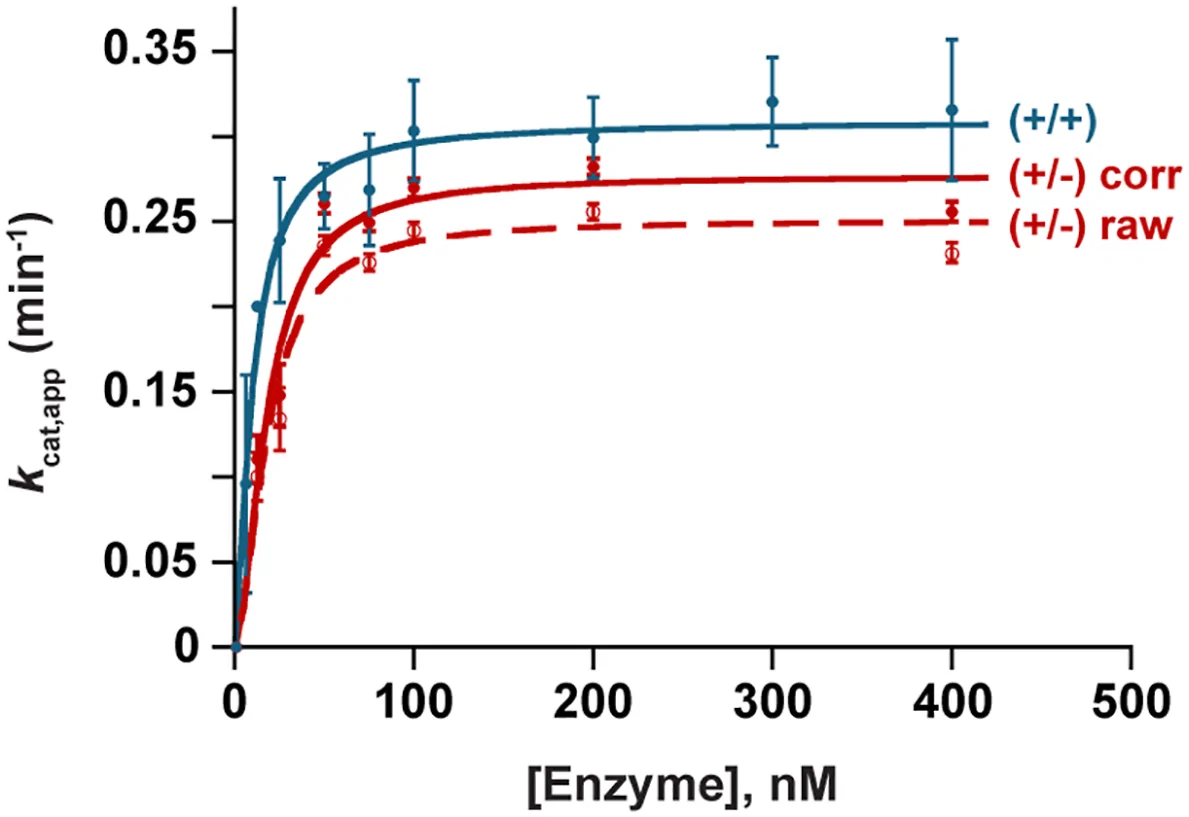
**Kinetic characterization of the (+/+) and (+/−) cePRMT1 heterodimers.** Activity was measured in the presence of 5 μM SAM and 20 μM H4(1-21) peptide at 22 °C. *Dasheddark red*: uncorrected (+/−) data (denoted with (+/−) raw), *solid dark red*: (+/−) data corrected for the percent heterodimer identified in solution by native MS (denoted with (+/−) corr), *dark blue*: (+/+) cePRMT1 (denoted with (+/+). Error bars represent standard deviation from the mean, and data points represent the average of at least two technical replicates.

Kinetic characterization of the (+/+) and (+/−) cePRMT1 enzymes was used to determine if PRMT1 displays half-of-sites reactivity. Consistent with previous studies that have used a kinetic approach to interrogate half-of-sites behavior (39, 40, 41), if cePRMT1 exhibits half-of-sites reactivity, we would expect the (+/+) and (+/−) to have similar maximal activity. If cePRMT1 does not exhibit half-of-sites reactivity, we would expect the (+/−) to have at most half (50%) of the maximal activity of the (+/+). Apparent k_cat_ (k_cat,app_) was measured as a function of enzyme concentration for both enzymes from 6.25 nM to 400 nM enzyme (Fig. 3). The substrate selected for methylation was a peptide consisting of the first 21 amino acids of the histone four protein (H4(1-21)). It is well established that PRMT1 oligomerization is concentration-dependent and that the monomeric form of PRMT1 is catalytically inactive, so maximum k_cat,app_ acts as a reporter for a fully dimeric enzyme population (32, 36). Comparing the maximum k_cat,app_ achieved by both constructs revealed that the (+/−) heterodimer exhibits ∼81% of the maximal activity of the (+/+) heterodimer. Considering that native MS showed that ∼90% of the species population was the (+/−) heterodimer, and that the remaining ∼10% of the species consists of the 2xstrep-tagged monomer and the 2xstrep-tagged homodimeric species that are unable to contribute to catalysis, the (+/−) heterodimer data was corrected by a factor of 1.1 (calculated from 100%/90%) to normalize the data and represent only the catalytically active heterodimeric fraction of the (+/−) enzyme preparation. A similar correction would not affect the kinetic data for the (+/+) enzyme since both subunits contribute to catalysis. With the correction, the (+/−) enzyme exhibits ∼90% of the activity of the (+/+) enzyme (Fig. 3). Through either analysis, the kinetic performance of the (+/−) heterodimer greatly exceeds the maximum 50% of activity expected for a whole-sites reactive enzyme and is far more consistent with half-of-sites reactivity. Importantly, this conclusion holds regardless of the correction, considering that if cePRMT1 were not half-of-sites reactive, a (+/−) enzyme population where 10% of the enzyme was inactive would be expected to have less than 50% of the (+/+) activity. Additionally, the (+/−) and (+/+) heterodimers were assayed in the presence of a saturating concentration of the protein substrate hnRNPK, and the (+/−) exhibited ∼97% of the activity of the (+/+) heterodimer (1.47 μM SAH∗s^−1^ for the (+/+) *versus* 1.42 μM SAH∗s^−1^ for the (+/−) with the correction), supporting that the model is consistent with half-of-sites reactivity and that this behavior is independent of the substrate being methylated.

### CePRMT1 binds H4(1-21) peptide and cofactor with a 1:1:2 dimer:peptide:cofactor stoichiometry along the reaction coordinate

**Figure 4:**
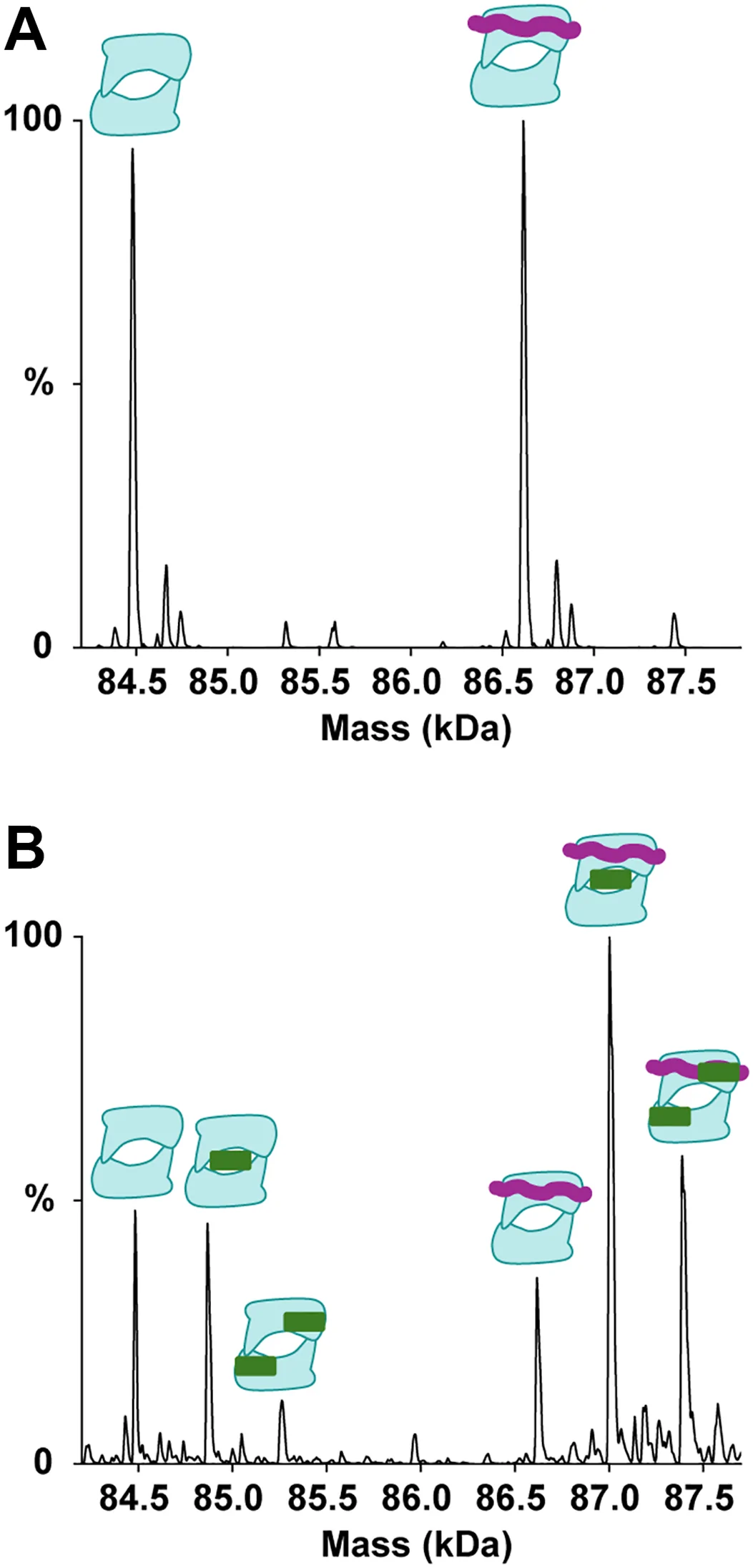
**CePRMT1 shows negative cooperativity for methylatable substrate binding in the presence and absence of SAM.** In all cases, proteins were rapidly solvent exchanged into 150 mM ammonium acetate adjusted to pH 7.6 prior to the addition of substrate and/or cofactor. *A*, deconvolved native MS spectrum for 1 μM (+/+) cePRMT1 with 20 μM H4(1-21). *B*, deconvolved native MS spectrum of cePRMT1 with 50 μM SAM and 20 μM H4(1-21). *Dark green*: SAH, *purple*: H4(1-21) peptide. The expected masses for each identified complex are available in Table S3.

To gain insight into *why* PRMT1 exhibits half-of-sites reactivity, native MS was used to determine substrate binding stoichiometry. Negative cooperativity of substrate binding is a common driving force of half-of-sites reactivity among homo-oligomeric enzymes and would involve a mechanism where PRMT1 binds methylatable substrate or cofactor in less than the total number of available binding sites (29, 43, 44, 45, 46). The mass shift for (+/+) cePRMT1 incubated with 20 μM H4(1-21) peptide alone was consistent with only one peptide being bound to the cePRMT1 dimer at a time (Figs. 4*A* and S4). Additionally, increasing the peptide concentration to 50 μM only resulted in minor second peptide binding (∼5%) (Fig. S8). Our results are consistent with observations from the Zheng group who determined a low μM binding event for peptide substrate followed by a second peptide binding event with a Kd ∼164 μM (47). Collectively, these data support that cePRMT1 exhibits negative cooperativity for methylatable substrate binding.

We considered the possibility that catalysis may influence cePRMT1 substrate or product stoichiometry. To address this, native MS was performed for (+/+) cePRMT1 incubated with 50 μM SAM prior to the addition of 20 μM methylatable H4(1-21) peptide, allowing catalysis to occur at the same substrate and enzyme concentrations used in our kinetic analyses. Cofactor-associated mass shifts show cePRMT1 being bound to either one or two molecules of SAH (+385 Da for each molecule). This is consistent with crystallographic and electron microscopy evidence that PRMT1 can bind SAH in both active sites within the dimer at the same time (PDB: 1ORI (30) and PDB: 8Z2Z (33)), and consistent with previous work demonstrating that each subunit in a human PRMT1 oligomer can independently bind SAM with identical affinity (47). However, peptide associated mass shifts were only consistent for a cePRMT1 dimer bound to a single H4(1-21) peptide, regardless of the presence or absence of cofactor bound to the enzyme (Fig. 4*B*). Additionally, evidence of methyl transfer can be identified at low *m*/*z*, at levels associated with free H4(1-21) in solution (Fig. S9). The observed 14 Da mass increase corresponds to the transfer of a single methyl group onto the H4(1-21) peptide, and we are able to observe up to two methyl transfer events. Overall, these data are consistent with a mechanism where a cePRMT1 dimer can mainly bind peptide in a single subunit during catalysis and supports that negative cooperativity for methylatable substrate binding drives half-of-sites reactive in cePRMT1.

### A cePRMT1 dimer containing a single functional active site can asymmetrically dimethylate arginyl residues

**Figure 5:**
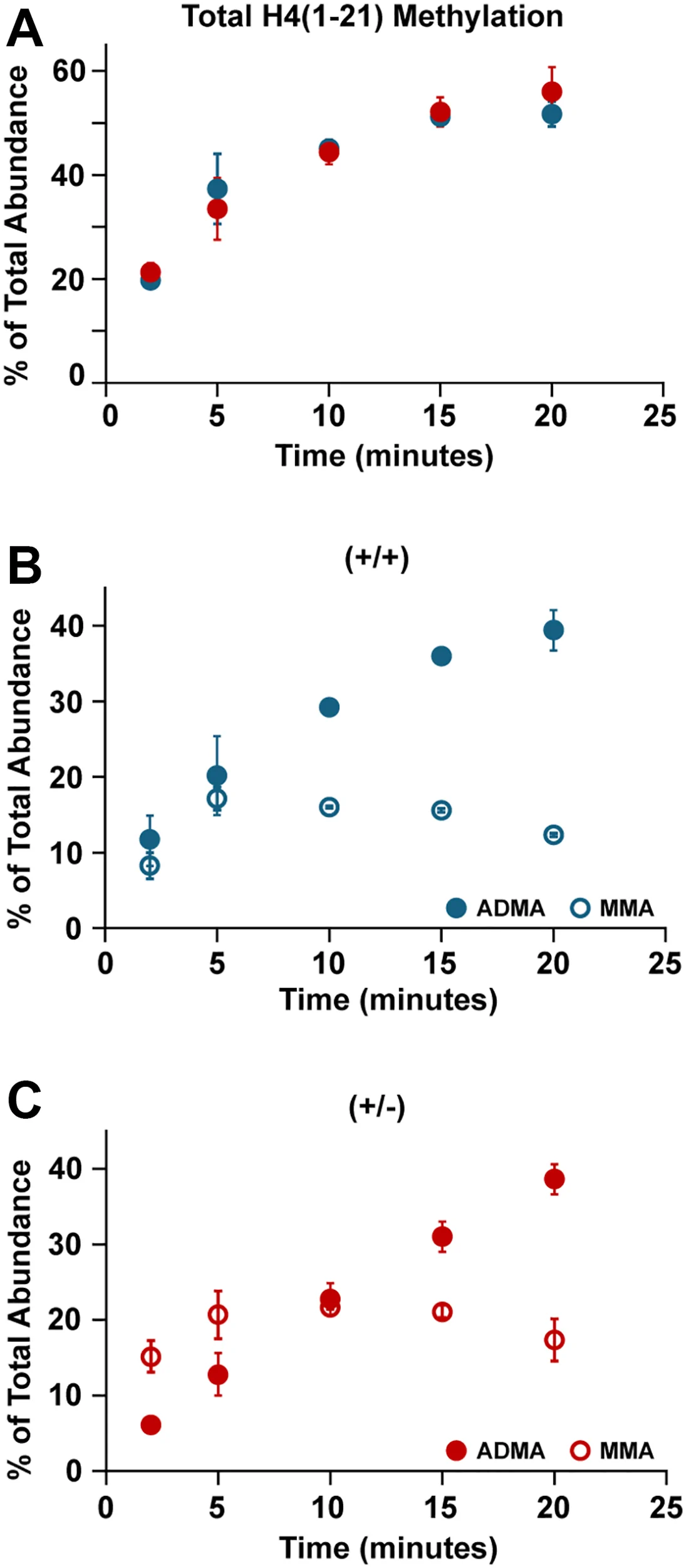
**A cePRMT1 dimer with a single functional active site can form ADMA.***A*, percent total abundance of ADMA and MMA combined for the (+/+) and normalized (+/−) enzymes. The percent total abundance for products and reactants measured for the (+/−) enzyme were normalized to that of the (+/+) enzyme by dividing by 0.81. *Dark red*: (+/−) cePRMT1, *blue*: (+/+) cePRMT1. *B*, percent total abundance of ADMA (*solid circle*) and MMA (*open circle*) for the (+/+) enzyme. *C*, normalized percent total abundance of ADMA (*solid circle*) and MMA (*open circle*) for the (+/−) enzyme. All points represent the average for two technical replicates, and error bars represent the standard deviation from the mean. Statistical significance between individual points was calculated using one-way ANOVA.

Our results demonstrate that individual subunits in a PRMT1 dimer communicate to regulate enzyme activity. To further characterize the role of inter-subunit communication, we investigated whether it serves to regulate the *type* of product formed by the enzyme as well. Given that our (+/−) cePRMT1 heterodimeric enzyme possesses a single catalytically functional active site, we are uniquely positioned to address whether a single functional active site can form ADMA, and if the MMA to ADMA formation ratio differs from that of a PRMT1 dimer containing two catalytically functional active sites. Differential product formation was measured for the (+/+) and (+/−) cePRMT1 enzymes by performing methyltransferase reactions restricted to the linear range of product formation, where the methylation state of the H4(1-21) peptide substrate was monitored over time using electrospray ionization (ESI) mass spectrometry (Fig. S10). Since kinetic characterization showed that the (+/−) enzyme reproducibly exhibits ∼81% of the total methylation exhibited by the (+/+) enzyme (Fig. 3), the amount of products made by the (+/−) enzyme was normalized to that of the (+/+) enzyme by dividing the percent total abundance for products and reactants measured for the (+/−) enzyme by 0.81 (Fig. 5*A*). Performing this normalization results in data points for the (+/+) and (+/−) enzymes that nearly superimpose each other, allowing for clear identification of differences in the relative amounts ADMA and MMA formation for each enzyme that are not obstructed by differences in total enzyme activity.

The (+/−) enzyme was capable of ADMA formation, showing that a single PRMT1 active site can form ADMA. Although visually the (+/+) and (+/−) enzymes appear to exhibit differential patterns of methylation (Fig. 5, *B* and *C*), one-way ANOVA analysis found that the differences between the % ADMA and % MMA abundance at t = 2 and 5 min for the (+/−) enzyme were not statistically significant (Fig. S11). (Fig. 5, *B* and *C*). Overall, these data suggest similar product formation between the (+/+) and (+/−) enzymes, and that the presence of only one functional active site in a PRMT1 dimer has little influence on product specificity.

## Discussion

Using computational and experimental methodologies, we have shown that asymmetry exists in a catalytically competent PRMT1 dimer that allows for allosteric regulation of enzymatic activity through half-of-sites reactivity. Our refined asymmetric model of dimeric human PRMT1 required a single bound GGRG peptide and two SAM molecules to achieve and maintain the activated catalytic configuration, suggesting a half-of-sites mechanism. We then validated the half-of-sites reactivity observed *in silico* by demonstrating that a *C. elegans* PRMT1 dimer with a single functional active site exhibits similar levels of activity as a wildtype cePRMT1 dimer. Additionally, we then showed that the half-of-sites behavior observed in cePRMT1 is driven by negative cooperativity for binding methyl group-accepting substrate. Finally, we showed that a cePRMT1 dimer with a single functional active site can indeed form ADMA, providing an answer to a long-standing question in the PRMT field.

Identifying the origin of PRMT1 allosteric communication at the atomic level will be instrumental in effectively leveraging half-of-sites reactivity to aid in the development of sophisticated, next-generation PRMT1 inhibitors. Our development of the active computational dimer presented in this study provides a physiological framework for refined examination of allostery in PRMT1. This model will enable us to reveal whether information transfer between subunits is mediated through conformational changes that allow for communication across the dimer interface, a mechanism that was previously shown to be enable positive allostery in PRMT1 (25). Notably, conformational changes that propagate negative cooperativity are a common trend across the enzymes that demonstrate half-of-sites behavior (29, 48). Additionally, the chemically competent model of the PRMT1 dimer will allow us to simulate the effects that post-translational modifications may have on inter-subunit allosteric signaling, as well as how communication pathways may be influenced by the cancer-associated mutations that we previously showed prevent PRMT1 dimer formation (31).

Our work prompts an interesting question of whether half-of-sites reactivity is a common mechanism among PRMTs. As pointed out by the Zheng group (47), mass spectrometry studies on human PRMT6 (49), and structural insight from a PRMT4 crystal structure (PDB: 5DWQ) (50) both support a scenario where methylatable substrate can only be bound to one dimeric active site at a time. This suggests a similar half-of-sites mechanism across the type I enzymes, however, whether half-of-sites reactivity is exhibited by type II and III PRMTs enzymes poses unique challenges to address experimentally. Addressing this will have relevant implications for isoform-specific targeting.

While half-of-sites reactivity is a recurring phenomenon among oligomeric enzymes (51), no unifying advantage has been identified to account for its conservation across diverse enzyme systems. Our (+/−) cePRMT1 exhibited close to 100% of the activity of the wildtype form of the enzyme, which suggests that a fully functional PRMT1 molecule can dimerize with a loss-of-function PRMT1 molecule and still achieve nearly full activation of a dimeric unit. This exposes an interesting parallel to the *Trypanosoma brucei* PRMT1, where the subunit that serves as the functional methyltransferase must first form a heterooligomer with a catalytically dead homolog before catalysis can occur (52). One hypothesis for this functionality in other oligomeric half-of-sites reactive enzymes is that it may serve as a protective mechanism by providing a “catalytic buffer” (53). Such a mechanism may allow for the cell to tolerate some amount of damaged or inactive subunits, such as those generated during oxidative stress or through mutagenesis. We also speculate that modification of one subunit may subsequently modulate enzymatic activity in another, possibly providing versatile and dynamic regulation of PRMT1 activity. Our design of a PRMT1 dimer with a single functional active site enables these ideas to be experimentally explored in future work.

## Experimental procedures

### Molecular dynamics simulations

All-atom MD simulations were performed using the AMBER22/AmberTools23 (54, 55) package to simulate hPRMT1 dimers bound with an GGRG peptide and two S-adenosylmethionine (SAM) substrates. The second-generation Generalized Amber Force Field (GAFF2) (56) and AmberTools antechamber package (57) were utilized to create the non-bonded and bonded parameters for SAM. Partial atomic charges were computed using restrained electrostatic potentials (RESP) (58). For RESP calculations, the structure of SAM was built using GaussView (59) and geometry-optimized using Gaussian16 (60) with the B3LYP/6-31+G(d) method (61, 62, 63, 64, 65) in conjunction with the Polarizable Continuum Model (PCM) (66) to simulate an aqueous environment. The structure of the PRMT1 dimer was adapted from a dimer subunit of a hPRMT1 tetramer (PDB entry 9BHG) (33) and protonated using the H++ server (http://biophysics.cs.vt.edu/H++) (67, 68, 69) using a temperature of 300 K and pH of 7.4. The acetyl/*N*-methylamide capped GGRG tetrapeptide was adapted from a GGRGG-docked PRMT1 system reported by Gathiaka *et al.* (35) by overlaying the structure with one of the dimer subunits using UCSF Chimera. The ff19SB (70) forcefield was used to parameterize both PRMT1 and the GGRG peptide.

All systems were charge neutralized using sodium ions and were solvated explicitly using the TIP3P water model (71) to a distance of 15 Å beyond the protein edge. Long-range electrostatic interactions were treated using the Particle-Mesh-Ewald (PME) method (72), utilizing a default cutoff range of 10 Å. The SHAKE (73) algorithm was used to constrain bonds involving hydrogen atoms, and the time-step for all simulations was set to 2 fs. All simulations were pre-minimized using a 5-step method: Step 1: restrain the PRMT1 dimer and substrates using a 10 kcal/mol harmonic bias, Step 2: reduce restraints to a 5 kcal/mol, Step 3: unrestrained the bound SAM, but keep restraints of 5 kcal/mol for PRMT1 dimer and GGRG peptide, Step 4: reduce restraints to 2 kcal/mol, and Step 5: remove all restraints entirely. All minimization steps used 2000 steepest descent cycles followed by 3000 conjugate gradient cycles using the GPU-enabled AMBER22 pmemd engines (74, 75). Equilibration using the NVT ensemble was performed for 2 ns followed by 8 ns of NPT equilibration using the Langevin thermostat (76) set to 300 K with a collision frequency of 1 ps^−1^ and the Berendsen barostat (73) set to 1 atm. Thermostat seeds were randomly generated using atmospheric noise (www.random.org).

For the restrained simulations, several harmonic restraints were utilized to anchor key hydrogen bonding residues between protein residues and SAM or GGRG substrate. These restraints were defined by hydrogen bonding pairs with donor-acceptor distances below 3.0 Å in the minimized structure. The reaction coordinate was also preserved with a 3.0 Å restraint between the SAM methyl carbon and the closest arginine guanidino nitrogen atom. Following equilibration, an additional 500 ns NPT equilibration was performed before the final unrestrained 500 ns NPT unbiased MD production trajectory. An exploratory asymmetric dimer simulation employed an alternative procedure that retained its active site restraints and utilized accelerated molecular dynamics (aMD) with a dihedral-only boost potential, using a threshold of 8000 kcal/mol and an acceleration α of 2650 kcal/mol. Three out of 10 clusters obtained *via* K-means clustering of the aMD trajectory were selected due to their *N*-terminus asymmetry. Cluster 1, Cluster 2, and Cluster five comprised of 26%, 17%, and 14% of production frames, respectively (Fig. S4). Representative pdb coordinates from these clusters were re-minimized and re-equilibrated following the above protocol before being subject to unrestrained, unbiased MD production runs. The viability of these clusters was ascertained by using the mean energy of the production trajectory compared to the mean energy of the asymmetric initial state trajectory (Table S2). Cluster two was selected as the representative form of the asymmetric PRMT1 dimer rearranged state as it alone had an energy below the predicted rearrangement energy.

Distances (Å) and angles (degrees) between SAM and arginine in GGRG in the PRMT1 dimer active site were calculated using cpptraj and used for a near-attack conformer analysis using a weighted distance function *d* + 0.5cos(θ). The attack distance *d* is defined as the distance between the transferred methyl carbon in SAM and one of the two nitrogen atoms (N_η1_ and N_η2_) in the arginine residue of the GGRG substrate (Fig. S1). The attack angle *θ* is defined as the angle between the SAM sulfur atom, the SAM methyl carbon, and either N_η1_ or N_η2_. For reference, typical active site transition states involving O, C, N and S atoms possess ideal reaction distances of around 3.2 Å and angles of ±15° of the transition state bonding angle (73). As such, an optimal S_N_2 attack *θ* of 180° at 3.2 Å would yield a final value of 2.7 Å in the reported histograms using the weighted equation. Near-attack conformer plots for the initial dimer conformation and the activated reactive complex are provided in Figures S3 and S4. Examination of the plots showed a much more idealized S_N_2 geometry in the activated complex with a *d* + 0.5cos(θ) of 2.75 Å with a probability height of 2.34 between SAM and N_η2_ compared to 3.40 Å and 1.08 for the initial PRMT1 dimer conformation (Table S1). Root mean square deviation (RMSD), root mean square fluctuation (RMSF), and distances between SAM and GGRG over the final 500 ns of the production trajectory of all three triplicate MD runs are provided in the Supporting Information (Figs. S12–S17).

### Plasmid generation

The wildtype sequence used for all *C. elegans* PRMT1 (cePRMT1) plasmids was obtained from the National Center for Biotechnology Information (NCBI) database (Reference sequence: NM_075508.8) and plasmids were commercially purchased through GenScript unless otherwise stated. To enable tandem expression of the his-tagged and 2xstrep-tagged subunits and immediate formation of his/2xstrep heterodimer, the (+/+) and (+/−) cePRMT1 plasmids were constructed using a pET DUET-1 vector. For the (+/+) construct, DUET Plasmid A was designed containing the Nco1-6Xhis-cePRMT1-Not1 DNA sequence inserted into multiple cloning site (MCS) I, and the NdeI-strep-strep-Tobacco Etch Virus protease cleavage site (TEV)-cePRMT1-Xho1 sequence inserted into MCS II. For the (+/−) construct, DUET Plasmid B was designed with an empty MCS I and the NdeI-strep-strep-TEV-cePRMT1-Xho1 DNA sequence in MCS II. To introduce the E153A mutation, site-directed mutagenesis was performed using the single cePRMT1 sequence in DUET Plasmid B as a template. The sequence of the Ndel-strep-strep-TEV-E153A cePRMT1-Xho1 was confirmed through whole-plasmid sequencing. Then to complete the construction of (+/−) cePRMT1, the original NdeI-strep-strep-TEV-cePRMT1-Xho1 sequence in DUET Plasmid A was excised from MCS II using restriction enzymes, and the Ndel-strep-strep-TEV-E153A cePRMT1-Xho1 sequence was inserted. The final DNA sequences for the complete (+/+) and (+/−) cePRMT1 plasmids were confirmed through whole-plasmid sequencing.

### Expression and purification of recombinant proteins

The (+/+) or (+/−) cePRMT1 plasmids were transformed into *Escherichia coli* NiCo21 (DE3) cells and then starter cultures were grown overnight (12–16 h) in Luria Broth (LB) media at 37 °C. 500 ml flasks of LB media were seeded with 5 ml of overnight culture and outgrown until reaching an optical density of 0.5 at 600 nm. Flasks were allowed to cool to room temperature and then expression was induced by adding a final concentration of 0.5 mM isopropyl β-d-1-thiogalactopyranoside (IPTG). The induced cultures were grown for an additional 18 h at 16 °C before cells were harvested by centrifugation, flash froze in liquid nitrogen, and stored at −20 °C.

To minimize heterodimer dissociation or potential subunit reshuffling, the protein purification protocol was designed to minimize duration and protein dilution. Specifically, 3 g of cell pellet expressing either (+/+) or (+/−) cePRMT1 was resuspended in a 1:10 cell mass-to-buffer ratio using lysis buffer (50 mM sodium phosphate, 1 mM EDTA, 1 mM DTT, pH 7.6). Cells were lysed by sonication, and the lysate was clarified by centrifugation. The soluble fraction was filtered with a 0.45-micron syringe filter unit (Millex) and then loaded into a 1 ml Strep-Tactin XT 4Flow affinity column (iba) at 0.5 ml/min. His-tagged homodimeric species and bulk impurities were removed by washing the column with 10 column volumes (CV) of lysis buffer at 1.0 ml/min. After washing, 1 ml HisTrap FF column (Cytiva) was connected to the bottom of the Strep-Tactin affinity column, and the protein was eluted directly onto the HisTrap column with Elution Buffer 1 (50 mM D-Biotin, 50 mM sodium phosphate, 1 mM DTT, pH 8.3) at 0.5 ml/min 2xstrep-tagged homodimeric species were removed by washing the column with 10 CV of lysis buffer at 1.0 ml/min. At this point, the remaining his/2xstrep heterodimeric species were eluted from the column with Elution Buffer 2 (500 mM imidazole, 50 mM sodium phosphate, 2 mM EDTA, 1 mM DTT, pH 7.6) at 1.0 ml/min. Subunit stoichiometry for each fraction was assessed by SDS PAGE, stained with Coomassie Blue, and band intensity was quantified with ImageLab 5.1 software using the lanes and bands tool. Elution fractions containing ≥ 1 mg/ml protein and a 1:1 his/2xstrep subunit band intensity were pooled and rapidly buffer exchanged into storage buffer (50 mM sodium phosphate, 1 mM EDTA, 5% v/v glycerol, 1 mM DTT, pH 7.6) with a 10 DG desalting column (BioRad) using the minimal dilution protocol suggested by manufacturer. Proteins were beaded in liquid nitrogen and stored at −80 °C. Concentration was assessed by measuring absorbance at 280 nm, and protein was stored at ≥ 1 mg/ml.

### Methyltransferase assays of cePRMT1

Quantifying (+/+) and (+/−) cePRMT1 methyltransferase activity over an enzyme concentration range of 6.25 to 400 nM was accomplished using the commercially available MTase-Glo kit from Promega. Briefly, reactions contained 20 mM Tris–HCl (pH 8.0), 50 mM NaCl, 3 mM MgCl_2_, 1 mM EDTA, 1 mM DTT, varied BSA to maintain a total protein concentration of 0.55 μM, 20 μM H4(1-21) peptide substrate, and 5 μM SAM. Reactions were initiated with an enzyme stock 20X the final concentration. Activity was measured at 22 °C because a decrease in activity was observed at 37 °C, similar to what has been observed for *C. elegans* PRMT5 (77). The methylation of 4 μM hnRNPK was accomplished using 400 nM enzyme and used 10 μM SAM. Reaction timepoints were chosen in the linear range of the reaction and were quenched in 0.5% TFA. The MTase-Glo reagent and MTase-Glo Detection reagents were added to each reaction and SAH standards following the manufacturer's protocol. Luminescence was detected with a BioTek luminometer; relative luminescence was converted to micromolar SAH using a SAH standard curve.

To ensure that the E153A mutation sufficiently abolished enzymatic activity, kinetics assays were performed with a strep-tagged homodimeric construct of the subunit containing the E153A mutation (−/− cePRMT1). Reactions contained 50 mM sodium phosphate pH 7.6, 1 mM DTT, 0.15 μM BSA, 10 nM MTAN (5′-methylthioadenosine/S-adenosylhomocysteine nucleosidase) to breakdown excess SAH (78), 20 μM H4(1-21) peptide substrate, and 50 μM total SAM, composed of 49 μM non-radioactive SAM, and 1 μM [H^3^] SAM (Revvity, specific activity: 84.5 Ci/mmol). Reactions were initiated with enzyme for a final concentration of 1 μM and proceeded at 22 °C. At different time points, 10 μl samples of the reaction were quenched in 12 μl of 8 M guanidinium hydrochloride with 2.5% TFA. Samples were processed with ZipTip_C18_ pipette tips (Millipore) for removal of unreacted [H^3^] SAM and recovery of the radiolabeled product as previously described (79). Radiolabeled product produced for each time point was quantified by a liquid scintillation counter.

Methyltransferase reactions designed for measuring differential product formation by ESI mass spectrometry contained 20 mM Tris–HCl (pH 8.0), 50 mM NaCl, 3 mM MgCl_2_, 1 mM EDTA, 1 mM DTT, 0.15 μM BSA, 5% (v/v) glycerol, 30 μM H4(1-21) peptide substrate, and 300 μM SAM. Reactions were initiated with a 20X enzyme stock for a final enzyme concentration of 1 μM enzyme, and proceeded at 22 °C. Time points were removed and were quenched in 7.5 μl of 100% (w/v) TCA for a final concentration of 5% TCA. The reactions were then deproteinized by incubating at 4 °C in an ice bath for 1 h and clarified through centrifugation at 12,000*g* for 30 min. The peptide solutions were diluted with 0.5 volumes of 400 mM sodium phosphate pH 7.6 to increase the pH to ∼2.0 before being desalted with C18 SpinTips (Pierce) and C18 Spin Columns (Pierce). The eluent for each timepoint was completely dried through vacuum centrifugation and stored at −80 °C. Each timepoint contained a total peptide mass of 10 μg. The linearity of this reaction was determined using the MTase-Glo kit (Promega).

### Native mass spectrometry

PRMT samples were buffer exchanged into 150 mM ammonium acetate (AmAc) (Sigma-Aldrich) using micro-Bio-spin P6 spin columns (BioRad). The pH was adjusted to 7.6 using ammonium hydroxide solution (Sigma-Aldrich) to match the kinetic assay conditions. Native MS experiments were performed on Thermo Q Exactive ultra high mass range (UHMR) Orbitrap instrument, which was modified with a surface-induced dissociation (SID) device between quadruple and C-Trap (80). PRMT WT (+/+) and (+/−) were diluted to 1 μM (dimer concentration) and immediately injected into the mass spectrometer. For PRMT-peptide substrate binding experiment, H4(1-21) peptide was added after PRMT buffer exchange to final concentrations of 20 or 50 μM. For PRMT–SAM–peptide reaction experiments, SAM was first added to PRMT at a final concentration of 50 μM and incubated at room temperature for 10 min. H4(1-21) peptide was then added immediately before MS analysis.

Samples were introduced into the MS instrument by nano-electrospray ionization, using glass capillary tips pulled in-house using a P-97 micropipette puller (Sutter Instruments). 3 to 5 μl of each sample was loaded into the tips, and the electrospray voltage was set between 0.5–1.0 kV. For intact enzyme complexes, the mass spectrometer was operated in positive mode using the following tuning settings: Source temperature: 250 °C; detector optimization: low m/z; ion transfer target: high m/z; in-source trapping (IST): 30 V; HCD trap gas flow: 5 to 7; Resolution: 12,500 or 25,000. For peptide alone, ion transfer target was set at low m/z, HCD trap gas flow was set at 3; while other tunings parameters were kept the same. Mass spectra were examined using Xcalibur 4.1 (Thermo Scientific), and deconvolved using UniDec software (81).

### Liquid chromatography-mass spectrometry analysis of peptide methylation

Liquid chromatography-tandem mass spectrometry (LC-MS/MS) was conducted using a trapping nano-flow LC-Orbitrap Astral MS system, which comprised a Dionex Ultimate 3000 nano LC system, a Dionex Ultimate 3000 gradient micro-LC system with a WPS-3000 autosampler, and an Orbitrap Astral mass spectrometer (Thermo Fisher Scientific).

Samples were first loaded onto a trapping column (150 μm ID × 5 cm, packed with 5 μm C18, CoAnn Technologies, Richland, WA), prior to separation on an OptiSpray μPAC Neo column (50 cm, 180 μm bed width, C18, Thermo Fisher Scientific, Waltham, MA). Mobile phases A and B were 0.1% formic acid (FA) in 2% acetonitrile, and 0.1% FA in 88% acetonitrile, respectively. The LC gradient profile was as follows: 4% to 34.1% B for 30 min; 34.1% to 97% B for 0.1 min; and isocratic at 97% B for 5.9 min.

Mass spectrometric data were acquired in data-dependent acquisition (DDA) mode. MS1 spectra were acquired in the m/z range of 200 to 1500 at a resolution of 120,000. The normalized Automatic Gain Control (AGC) target for MS1 was set at 500%, with a maximum injection time of 5 ms. Dynamic exclusion was enabled with a duration of 10 s. Precursor ions were isolated using a 1.6 Th isolation window and fragmented by higher-energy collision-induced dissociation (HCD) at a normalized collision energy of 30%. MS2 spectra were acquired in the Astral mass analyzer. The normalized Automatic Gain Control (AGC) target was set to standard with a maximum injection time of 22 ms.

Raw MS files were analyzed in Thermo Proteome Discoverer, version 3.1.0.638, using the Sequest HT search engine. Spectra were searched against a custom database containing only the H4(1-21) peptide sequence. The database search was performed using a no-enzyme (unspecific) digestion setting. Peptide N-terminal acetylation and peptide C-terminal biotinylation were specified as static modifications, whereas mono- and dimethylation of arginine residues were specified as variable modifications. The precursor and fragment ion mass tolerances were set to 20 ppm and 0.02 Da, respectively. Peptide-spectrum matches were filtered using the Fixed Value PSM Validator with a maximum delta Cn of 0.05. Because the search was restricted to a custom database containing the H4(1-21) peptide sequence, peptide-spectrum matches were evaluated using these workflow-based criteria rather than global target-decoy FDR estimation. Precursor ion abundances were quantified using the Minora Feature Detector node.

### Native PAGE

Native PAGE experiments were performed as described previously (32). Briefly, enzymes were buffer exchanged into 50 mM sodium phosphate (pH 7.6), 1 mM DTT, 1 mM EDTA, and 5% glycerol using a Zeba spin column (Thermo Fisher), and then diluted further to a final concentration of 600 nM prior to loading. Human PRMT1 was included as a protein standard for tetrameric PRMT1. Samples were mixed and allowed to incubate at 25 °C for 15 min then run on a 4%–20% TGX Mini-PROTEAN Precast Protein Gel (BioRad) at 100 V for 10 h at 4 °C. To visualize protein, the gel was stained with 1X SYPRO orange protein stain (Invitrogen) in 7.5% (v/v) acetic acid for 45 min at room temperature and briefly washed with 7.5% (v/v) acetic acid immediately before imaging for fluorescence.

## Data availability

The datasets used and/or analyzed during the current study are available from the corresponding author on reasonable request.

## Supporting information

This article contains supporting information (36, 82).

## Conflict of interest

The authors declare that they have no conflicts of interest with the contents of this article.
