# Supplementary material for: Negative allosteric regulation of protein arginine methyltransferase 1 (PRMT1) through half-of-sites reactivity
Source: J Biol Chem. 2026 Aug 12;302(10):113425. doi: 10.1016/j.jbc.2026.113425 (PMC13571421; doi:10.1016/j.jbc.2026.113425)
Supplement: Supplementary file 1 [file mmc1.docx]

Negative allosteric regulation of Protein arginine methyltransferase 1 (PRMT1) through half-of-sites reactivity

Vincent Z Rossi ^1^, Robert S. Wilson-Kovacs^2^‡, Yuan Gao^3^‡, Caroline Velez^2^, Sofiia Hakh^1^, Chao Xue^4^, Jun Qu^4^, Vicki Wysocki^3^, Orlando Acevedo^2^, and Joan M Hevel^1^*

^1^Department of Chemistry and Biochemistry, Utah State University, Logan, Utah, USA

^2^Department of Chemistry, University of Miami, Coral Gables, Florida, USA

^3^School of Chemistry & Biochemistry, Georgia Institute of Technology, Atlanta, Georgia, USA

^4^Department of Pharmaceutical Sciences, University at Buffalo, Buffalo, New York, USA

‡These two authors contributed equally to the manuscript

* To whom correspondence should be addressed

**Supporting Information**

**Figure S1.** S_N_2 methyltransferase reaction definition

**Table S1.** Near-attack conformer analysis maximum peak probabilities and weighted reacting distances (Å) for the initial state and activated asymmetric PRMT1 dimer

**Figure S2.** Probability of sampling near-attack conformers in the initial PRMT1 asymmetric dimer complex that leads to the formation of monomethyl arginine (MMA) product

**Table S2.** The relative energies, Δ*E* (kcal/mol), of representative structures of aMD clusters compared to the PRMT1 asymmetric dimer ground state calculated from their production trajectories

**Figure S3.** Probability of sampling near-attack conformers in the activated catalytic PRMT1 asymmetric dimer complex that leads to the formation of monomethyl arginine (MMA) product

**Figure S4.** Representative structures of PRMT1 clusters obtained via aMD

**Figure S5.** Native PAGE demonstrates *C.elegans* PRMT1 migrates as a dimer

**Figure S6.** Loss-of-function mutation E153A abolishes activity of a dimer with the E153A mutation in both active sites (-/- cePRMT1)

**Figure S7.** Uncropped 10% SDS PAGE gels for heterodimer subunit quantification

**Figure S8.** nMS spectra for cePRMT1 alone, and with 20 or 50 µM H4(1-21)

**Figure S9.** Low m/z peptide-level detection of H4(1-21) methylation by cePRMT1

**Table S3.** Expected masses for the enzyme-substrate complexes identified by native MS

**Figure S10.** Total percent of product abundance measured by ESI mass spectrometry (no normalization)

**Figure S11.** Percent total abundance of ADMA and MMA for the (+/+) and normalized (+/-) enzymes

**Figure S12.** All-atom RMSD calculations of triplicate simulations of the activated catalytic PRMT1 asymmetric dimer complex

**Figure S13.** Reaction coordinate distance between the nitrogen atom in arginine and the methyl carbon in SAM located within the activated catalytic PRMT1 asymmetric dimer active site for each simulation

**Figure S14.** All-atom RMSF calculations of triplicate 1 of the activated catalytic PRMT1 asymmetric dimer complex, relative to their initial frame

**Figure S15.** All-atom RMSF calculations of triplicate 2 of the activated catalytic PRMT1 asymmetric dimer complex, relative to their initial frame

**Figure S16.** All-atom RMSF calculations of triplicate 3 of the activated catalytic PRMT1 asymmetric dimer complex, relative to their initial frame

**Figure S17.** All-atom RMSF calculations of the GGRG peptide substrate located within the activated catalytic PRMT1 asymmetric dimer active site, relative to their initial frame

**Figure S1.** S_N_2 reaction definition where *d* is the distance (Å) between the SAM methyl carbon and the N_η1_ or N_η2_ of the GGRG arginine and *θ* is the angle (degrees) between the SAM sulfur, the SAM methyl carbon and N_η1_ or N_η2_.

**Table S1.** Near-attack conformer analysis maximum peak probabilities and weighted reacting distances (Å) for the initial state and activated asymmetric PRMT1 dimer.

|  | *d* + 0.5cos(θ) | Max peak probability |
| --- | --- | --- |
| Initial N_η1_ | 4.15 | 1.12 |
| Initial N_η2_ | 3.40 | 1.08 |
| Activated N_η1_ | 3.46 | 1.61 |
| Activated N_η2_ | 2.75 | 2.34 |

**Figure S2.** Probability of sampling near-attack conformers in the initial PRMT1 asymmetric dimer complex that leads to the formation of monomethyl arginine (MMA) product. The preferred orientations by the substrate over time were evaluated by comparing the distributions of *d* + 0.5cos(θ) (in Å) for all trajectories, where *d* is the attack distance between the nitrogen atom in arginine (N_η1_ and N_η2_) and the methyl carbon in SAM, and θ is the respective attack angle (N^…^CH_3_^…^S).

**Table S2.** The relative energies, Δ*E* (kcal/mol), of representative structures of aMD clusters compared to the PRMT1 asymmetric dimer ground state calculated from their production trajectories. For reference, an estimated activation energy for the protein transition was deduced using the kinetic model of Feng et al. (36) and calculated using the Eyring equation (82) using a typical protein transmission coefficient of 0.1.

| **Protein State** | **ΔE (kcal/mol)** |
| --- | --- |
| Initial State | 0 |
| Cluster 1 | 31.1 |
| Cluster 2 | 10.6 |
| Cluster 5 | 30.4 |
| Rearrangement Energy | 17.4 |

(36) Feng, Y. et al. *A Transient Kinetic Analysis of PRMT1 Catalysis.* *Biochemistry*, **2011**, *50*, 7033-7044. (82) Eyring, H. *The Activated Complex in Chemical Reactions.* *J. Chem. Phys*., **1935**, *3*, 107–115.

**Figure S3.** Probability of sampling near-attack conformers in the activated catalytic PRMT1 asymmetric dimer complex that leads to the formation of monomethyl arginine (MMA) product. The preferred orientations by the substrate over time were evaluated by comparing the distributions of *d* + 0.5cos(θ) (in Å) for all trajectories, where *d* is the attack distance between the nitrogen atom in arginine (N_η1_ and N_η2_) and the methyl carbon in SAM, and θ is the respective attack angle (N^…^CH_3_^…^S).

**Figure S4.** Representative structures of PRMT1 clusters obtained via aMD. The GGRG peptides are shown in red, SAM substrate in green, THW loop in purple, and the N-termini in black.

**Figure S5**. Native PAGE demonstrates *C.elegans* PRMT1 migrates as a dimer. Human PRMT1 was included as a protein standard for tetrameric PRMT1.

**Figure S6**. Loss-of-function mutation E153A abolishes activity of a dimer with the E153A mutation in both active sites (-/- cePRMT1). To ensure that the E153A mutation sufficiently abolished enzymatic activity, kinetics assays were performed with a strep-tagged homodimeric construct of the subunit containing the E153A mutation (-/- cePRMT1) and demonstrate that the catalytically dead subunit does not contribute more than 1% of the total activity expected by the (+/-) heterodimer (**Figure 2D**). Assays were performed in the presence of 1 µM enzyme, 20 µM H4(1-21) peptide, and 50 µM SAM at 22°C. Bars represent the average for two technical replicates, and error bars represent the standard deviation from the mean.

**Figure S7**. Uncropped 10% SDS PAGE gels for heterodimer subunit quantification. The BLUEstain™ Protein Ladder (GoldBio) was used for molecular weight standards under tris-glycine running conditions. **A)** (+/-) cePRMT1. **B)** (+/+) cePRMT1.

**Figure S8.** nMS spectra for cePRMT1 **A)** alone, **B)** with 20 μM H4(1-21), and **C)** with 50 μM H4(1-21). nMS shows cePRMT1 dimer only able to binds mostly up to 1 H4(1-21) copies. Even at 50 μM H4(1-21), the two binding species are neglectable (about 5%), consistent with negative cooperativity of H4(1-21) binding.

**Figure S9.** Low m/z peptide-level detection of H4(1-21) methylation by cePRMT1. After incubation with cePRMT1 WT and SAM, new peaks corresponding to +14 Da and +28 Da mass shifts are observed, consistent with the addition of one and two methyl groups to the H4(1-21) peptide, respectively.

**Table S3.** Expected masses for the enzyme-substrate complexes identified by native MS that are presented in **Figure 4**. The (+/+) cePRMT1 enzyme was used for these experiments.

| Identified Complex | Expected Mass (kDa) |
| --- | --- |
| 2 cePRMT1 | 84.747 |
| 2 cePRMT1 + 1 SAH | 85.131 |
| 2 cePRMT1 + 2 SAH  2 cePRMT1 + 1 H4(1-21) | 85.515  86.880 |
| 2 cePRMT1 + 1 SAH + 1 H4(1-21) | 87.264 |
| 2 cePRMT1 + 2 SAH + 1 H4(1-21) | 87.648 |

**Figure S10**. Total percent of product abundance measured by ESI mass spectrometry. Blue: (+/+) cePRMT1, dark red: (+/-) cePRMT1 (not normalized to the activity of the (+/+) enzyme). Points represent the average for two technical replicates, and error bars represent the standard deviation from the mean.

**Figure S11.** Percent total abundance of ADMA and MMA for the (+/+) (shown in blue) and normalized (+/-) enzymes (shown in dark red). The data for the (+/-) enzyme was normalized to that of the (+/+) enzyme by dividing by 0.81. **A)** total percent H4(1-21) dimethylation and **B)** total percent H4(1-21) monomethylation. Points represent the average for two technical replicates, and error bars represent the standard deviation from the mean.

**Triplicate 1**

**Triplicate 2**

**Triplicate 3**

**Figure S12.** All-atom RMSD calculations of triplicate simulations of the activated catalytic PRMT1 asymmetric dimer complex, relative to their initial frame.

**Triplicate 1**

**Triplicate 2**

**Triplicate 3**

**Figure S13.** Reaction coordinate distance between the nitrogen atom in arginine and the methyl carbon in SAM located within the activated catalytic PRMT1 asymmetric dimer active site for each of the triplicate simulations.

**Figure S14.** All-atom RMSF calculations of triplicate 1 of the activated catalytic PRMT1 asymmetric dimer complex, relative to their initial frame. α-helix (α_x_, E, G, I) and β-sheet (15) domains containing >2.0 Å RMSF residues are highlighted.

**Figure S15.** All-atom RMSF calculations of triplicate 2 of the activated catalytic PRMT1 asymmetric dimer complex, relative to their initial frame. α-helix (α_x_, E, G, I) and β-sheet (15) domains containing >2.0 Å RMSF residues are highlighted.

**Figure S16.** All-atom RMSF calculations of triplicate 3 of the activated catalytic PRMT1 asymmetric dimer complex, relative to their initial frame. α-helix (α_x_, E, G, I) and β-sheet (15) domains containing >2.0 Å RMSF residues are highlighted.

**Figure S17:** All-atom RMSF calculations of the GGRG peptide substrate located within the activated catalytic PRMT1 asymmetric dimer active site, relative to their initial frame.
